# Mouse t-complex protein 11 is important for progressive motility in sperm^†^

**DOI:** 10.1093/biolre/ioz226

**Published:** 2019-12-14

**Authors:** Julio M Castaneda, Haruhiko Miyata, Denise R Archambeault, Yuhkoh Satouh, Zhifeng Yu, Masahito Ikawa, Martin M Matzuk

**Affiliations:** 1 Department of Experimental Genome Research, Research Institute for Microbial Diseases, Osaka University, Osaka, Japan; 2 Department of Pathology and Immunology, Baylor College of Medicine, Houston, Texas, USA; 3 Department of Molecular and Cellular Biology, Institute for Molecular and Cellular Regulation, Gunma University, Gunma, Japan; 4 Center for Drug Discovery, Baylor College of Medicine, Houston, Texas, USA; 5 Immunology Frontier Research Center, Osaka University, Osaka, Japan; 6 Graduate School of Medicine, Osaka University, Osaka, Japan and; 7 School of Pharmaceutical Sciences, Osaka University, Osaka, Japan

**Keywords:** spermatogenesis, sperm motility, male fertility, t-complex protein, TCP11, spermiogenesis

## Abstract

The *t*-complex is defined as naturally occurring variants of the proximal third of mouse chromosome 17 and has been studied by mouse geneticists for decades. This region contains many genes involved in processes from embryogenesis to sperm function. One such gene, t-complex protein 11 (*Tcp11*), was identified as a testis-specific gene whose protein is present in elongating spermatids. Later work on *Tcp11* localized TCP11 to the sperm surface and acrosome cap and implicated TCP11 as important for sperm capacitation through the cyclic AMP/Protein Kinase A pathway. Here, we show that TCP11 is cytoplasmically localized to elongating spermatids and absent from sperm. In the absence of *Tcp11*, male mice have severely reduced fertility due to a significant decrease in progressively motile sperm; however, *Tcp11*-null sperm continues to undergo tyrosine phosphorylation, a hallmark of capacitation. Interestingly, null sperm displays reduced PKA activity, consistent with previous reports. Our work demonstrates that TCP11 functions in elongated spermatids to confer proper motility in mature sperm.

## Teaser

T-complex protein 11, Tcp11, encodes a cytoplasmic protein expressed in the late stages of spermiogenesis that is important for sperm motility.

## Introduction

The *t*-complex consists of the proximal third of mouse chromosome 17 that was initially discovered almost 90 years ago from crosses of tailless mice using wild and laboratory mouse strains [1, 2]. This region spans >35 MB and contains four tandem inversions that prevent recombination between the corresponding wild-type allele [3–7]. Homozygosity for a *t*-haplotype is usually lethal; however, different haplotypes may complement each other [6, 8–11]. Interestingly, these haplotypes exhibit transmission ratio distortion in that heterozygous males transmit the *t*-haplotype at a greater frequency than the wild-type version of this region [12]. While the exact mechanism of the transmission distortion has yet to be elucidated, there is evidence that the *t*-region encodes a responder and a distorter that increase the likelihood of sperm carrying the *t*-allele fertilizing eggs [12–16].

Within the *t*-complex region, the major histocompatibility complex is encoded along with other genes essential for embryogenesis [8, 17]. Several genes within the complex were also shown to be expressed during spermatogenesis and affect male fertility [18]. One of these genes at the distal end of the t-complex that exhibits testis-specific expression is t-complex protein 11 (*Tcp11*). *Tcp11* was identified through a differential hybridization screen of testis cDNAs [19]. It was later shown that TCP11 localizes to late stage spermatids as a soluble, non-membrane bound protein [20]. Interestingly, TCP11 was suggested to be the receptor for fertilizing promoting peptide, a tripeptide produced by the prostate gland in many mammalian species, and to function in sperm capacitation [21, 22]. More recently, TCP11 was shown to form a complex with PKA in sperm and to help regulate capacitation through the cAMP pathway [23].

Here, we show that genetic ablation of *Tcp11* produces subfertile male mice with sperm showing reduced motility. Through in vitro fertilization (IVF), *Tcp11*-null sperm are capable of fusing with zona pellucida-free oocytes but not with cumulus intact and cumulus free oocytes. Interestingly, IVF failure of *Tcp11*-null sperm can be partially rescued with glutathione treated oocytes. Examination of phosphotyrosine in the null sperm showed no obvious changes, further suggesting that *Tcp11* is essential for sperm motility and not for capacitation. PKA activity appears to be reduced in *Tcp11*-null sperm consistent with a role of TCP11 in the cAMP/PKA pathway.

## Results

### TCP11 is an evolutionary-conserved protein expressed in testis

**Figure 1:**
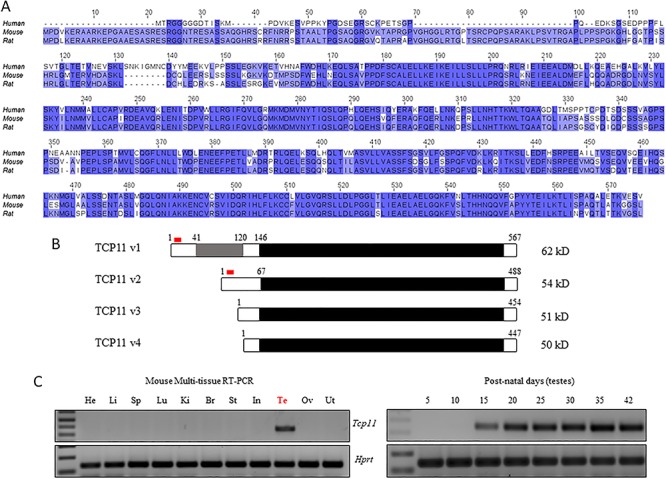
Tcp11 is evolutionary conserved and expressed in testis. (A) Multiple sequence alignment of human, mouse, and rat TCP11. Darker color represents greater conservation. (B) Two verified variants of mouse TCP11 are annotated, one 567 amino acids long and a shorter version 488 amino acids long. Two additional TCP11 variants are predicted that are shorter. Most of TCP11 is composed of the TCP11 domain (black), which is specific to TCP11 homologs and is uncharacterized. Grey highlights the region specific to variant 1. Red highlights the region used to generate an antibody. (C) RT-PCR from various mouse tissues detects expression of *Tcp11* in testis beginning at post-natal day 15. *Hprt* (hypoxanthine-guanine phosphoribosyltransferase) was used as a control.

*Tcp11* is evolutionary conserved amongst most metazoans and contains an uncharacterized protein domain, the TCP11 domain, that comprises most of the protein (Figure 1A). In mouse, *Tcp11* encodes two verified isoforms: the longer isoform consists of 567 amino acids (UniProt: B2KF24), while the shorter isoform is 488 amino acids (UniProt: Q5FWA2) (Figure 1B). The shorter isoform lacks 79 amino acids near the N-terminus. There are also two predicted isoforms at 454 and 447 amino acids long (UniProt: A0A3B2W7H0 and A0A3B2WD68, respectively) (Figure 1B). Reverse transcription polymerase chain reaction (RT-PCR) analysis from various mouse tissues confirms *Tcp11* as testis-specific, with expression in the testis commencing at post-natal day 15 when the leading edge of the first wave of spermatogenesis enters the pachytene stage (Figure 1C). In contrast to the mouse, RT-PCR detects human *TCP11* in the brain and epididymis in addition to strong expression in the testis (Supplementary Figure S1). *Tcp11* has undergone gene duplication in metazoans, with three paralogs present in mouse. *Tcp11x2* (*Tcp11l3*) is present on the X-chromosome while *Tcp11l1* and *Tcp11l2* are on chromosome 2 and 10, respectively (Supplementary Figure S2A). Homology between the mouse TCP11 paralogs ranges from 32 to 55% identity (Supplementary Figure S2B). All paralogs contain the TCP11 domain (Supplementary Figure S2C). RT-PCR shows that *Tcp11x2* is also testis-specific with *Tcp11l1* and *Tcp11l2* having broader expression (Supplementary Figure S2D).

### *Tcp11* knockout males are subfertile

**Figure 2:**
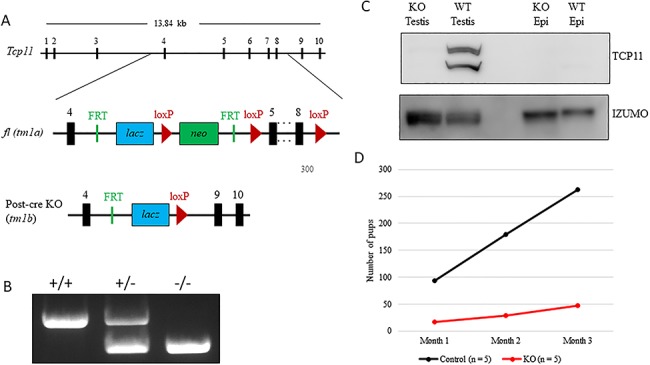
*Tcp11*-null males are subfertile. (A) Schematic of mouse *Tcp11*, the targeted *Tcp11^tm1a(WTSI)^* floxed allele (*Tcp11^fl^*), and the post-cre null allele *Tcp11^tm1b^* (*Tcp11^−^*). (B) Genotyping PCR distinguishing wild-type, heterozygous *Tcp11^+/−^*, and *Tcp11* knockout (*Tcp11^−/−^*) mice. (C) Western blot analysis with an antibody raised against a region near the N-terminus, detects both the longer and shorter variants of TCP11 in wild-type testis lysate but not in epididymal lysates. IZUMO1 (a component of the acrosome) confirms the presence of spermatozoa in both the testis and epididymal lysate. (D) Mating tests show *Tcp11^−/−^* sire fewer pups over a three-month mating period than controls when paired with wild-type females (*n* = 5 males).

To examine the role of *Tcp11*, we obtained mice carrying a knockout-first, conditional ready allele of *Tcp11* (*Tcp11^tm1a(WTSI)^*, referred to as *Tcp11^fl^*) [24, 25] and maintained the line on a mixed genetic background (Figure 2A). To obtain the post-cre null allele (*Tcp11^tm1b^*, referred to as *Tcp11^−^*), we crossed *Tcp11^fl/+^* mice with transgenic mice expressing iCre under the *Gdf9* promoter [26] to produce heterozygous offspring that were intercrossed to obtain full-body knockouts. The WTSI allele targets exons 5–8 for deletion. From the annotated mouse genome, there is a processed pseudogene between exon 4 and 5 (4930526A20Rik) within the *Tcp11* locus that encodes a tRNA splicing endonuclease. PCR genotyping readily distinguishes between the wild-type and tm1b allele (Figure 2B). *Tcp11^fl/+^* or *Tcp11^−/+^* mice were intercrossed to generate homozygous males. To confirm that *Tcp11^−/−^* is a true null allele, we generated an antibody to amino acids corresponding to 15–32 of mouse TCP11. Western blot analysis shows the longer and shorter TCP11 isoforms running at approximately 62 and 54 kD, respectively (Figure 2C and Supplementary Figure S3A). *Tcp11^−/−^* testis lysate did not reveal expression of TCP11, indicating that the *Tcp11^−/−^* mice are true knockout (KO; null) mice. Also, TCP11 was not detected in RIPA protein extracts from the epididymis, indicating that TCP11 is not present in mature spermatozoa (Figure 2C and Supplementary Figure S3A). We were able to obtain a previously published anti-TCP11 antibody raised against full-length mouse TCP11 [20, 21]; western blot analysis with this antibody detects the longer (62 kD) and shorter TCP11 (54 kD) as well as a doublet running above 50 kD and possibly a shorter fifth isoform confirming four of the predicted TCP11 isoforms (Supplementary Figure S3C and D). A commercially available anti-TCP11 antibody was also tested in testis and epididymal lysates from wild type and Tcp11-nulls and showed several non-specific bands (Supplementary Figure S3E). To test whether *Tcp11* might function in male fertility, we paired individual homozygous males with wild-type females (mixed genetic background) for three months and recorded the number of pups delivered. Fertility tests showed that the *Tcp11^−/−^* (*n* = 5) mice were subfertile siring fewer pups compared to control males (*n* = 5), demonstrating that TCP11 plays an important role in male fertility (Figure 2D).

### *Tcp11*-null sperm have decreased motility

**Figure 3:**
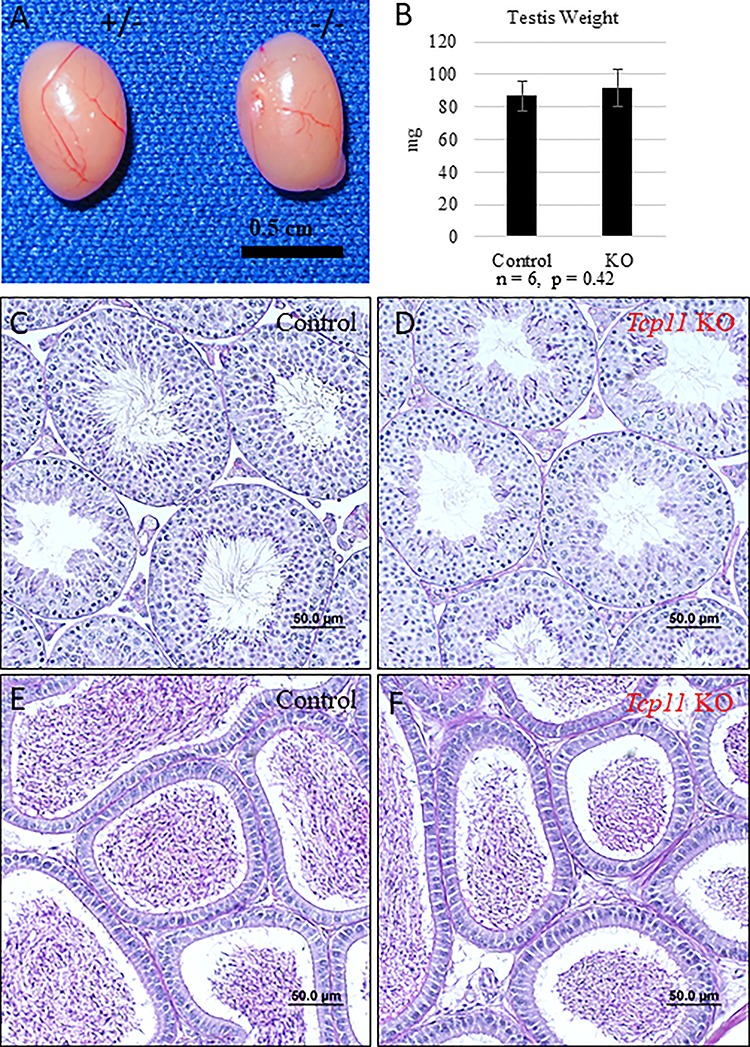
Spermatogenesis appears normal in the absence of *Tcp11*. (A) Gross morphology of testis from control (het) and *Tcp11* KO males. (B) Average testis weight from control and *Tcp11* KO adult males. (C and D) PAS stained testis cross sections from control and *Tcp11* KO mice, respectively. Scale bars = 50 μm. (E and F) PAS stained epididymal sections from control and *Tcp11* KO mice, respectively. Scale bars = 50 μm.

**Figure 4:**
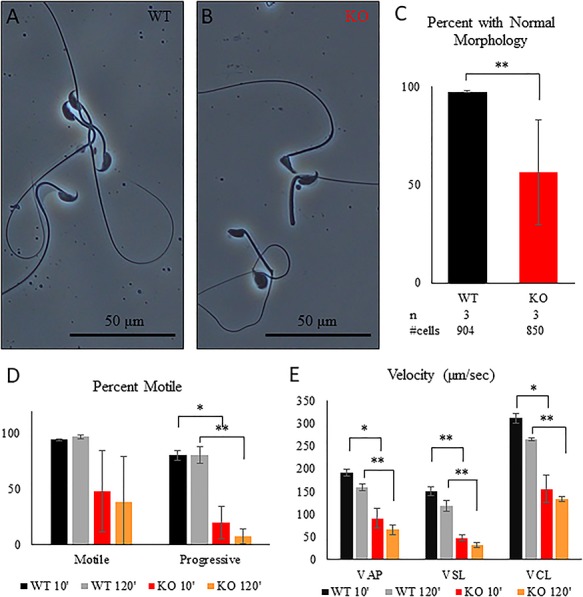
Tcp11 KO sperm exhibit aberrant morphology and reduced motility. (A and B) Phase contrast microscopy images of sperm isolated from the cauda epididymis from wild type and *Tcp11* KO. (C) Percent of sperm isolated from the cauda epididymis displaying abnormal morphology between wild type and *Tcp11* KO. (D) Percent of sperm that are motile and progressively motile in wild type and *Tcp11* KO determined by CASA analysis after incubation in TYH medium for 10 and 120 min. (E) Motility parameters of sperm from wild type and *Tcp11* KO determined by CASA analysis after incubation in TYH medium for 10 and 120 min. (^*^0.05 > *P* > 0.001; ^**^P < 0.001).

To determine the cause of the subfertility in *Tcp11^−/−^* male mice, we first examined spermatogenesis. The gross morphology is comparable, and no significant differences in testis weight were observed between adult control (heterozygous) and KO males (Figure 3A and B). Also, PAS stained testis and epididymis sections did not show noticeable differences between the two genotypes, suggesting no obvious defect in spermatogenesis (Figure 3C–F and Supplementary Figure S4B). Closer examination of spermatogenesis with immunofluorescence also did not reveal obvious differences between control and KO (Supplementary Movies S1–S4). Quantification of step 15–16 spermatozoa in seminiferous tubules (epithelial cycle VI–VIII) slightly more spermatozoa produced by the KO compared to the control (Supplementary Figure S4C). To examine sperm quality, sperm from the cauda epididymis was isolated into TYH medium and incubated at 37 °C with 5% CO_2_ for 10 and 120 min. After incubation in TYH medium, we visualized sperm morphology and utilized Computer Assisted Sperm Analysis (CASA) to examine sperm quality. Phase contrast microscopy revealed aberrantly shaped sperm heads in sperm examined from the KO mice when compared with wild-type mice (Figure 4A and B). Quantification revealed a significant decrease of morphologically normal sperm in the KO (Figure 4C). CASA analysis not only revealed a decrease in the percent of motile sperm in the KO, but also all velocity parameters of sperm motility (average path velocity, straight line velocity, and curvilinear velocity) were decreased (Figure 4E and F and Supplementary Movies 5 and 6). These data indicate that TCP11 plays a role in sperm motility.

**Figure 5:**
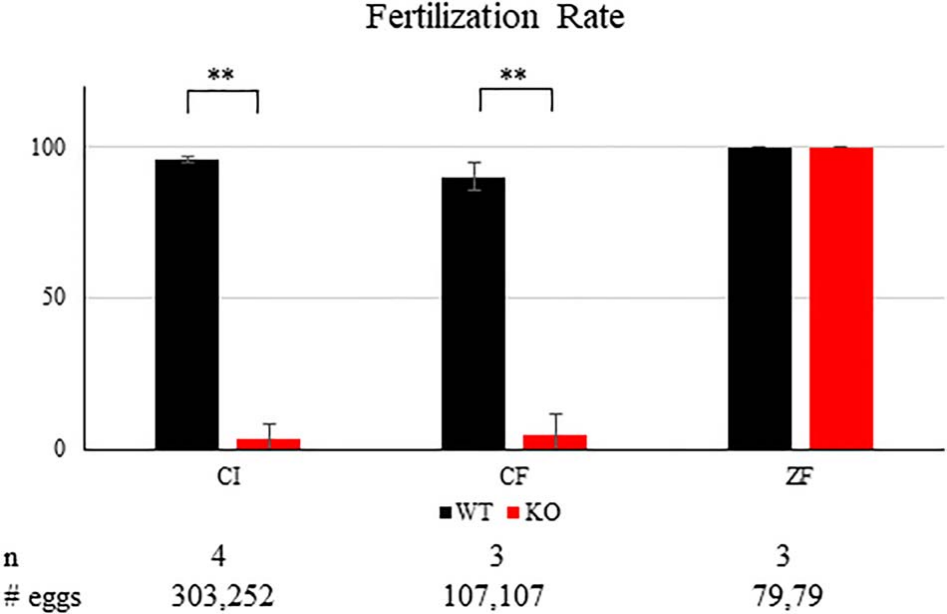
Tcp11 KO sperm can fertilize oocytes without the zona pellucida. Fertilization rates using wild-type or *Tcp11* KO sperm obtained from the cauda epididymis. CI, cumulus intact; CF, cumulus free; ZF, zona free (^**^P < 0.001).

To pinpoint which step in fertilization *Tcp11*-null sperm encounter problems, we carried out IVF. *Tcp11*-null sperm isolated from the cauda epididymis fertilized few cumulus-intact and cumulus-free oocytes but efficiently fertilizes zona pellucida-free oocytes (Figure 5). Successful IVF with zona pellucida-free eggs demonstrates that TCP11 does not function in sperm-egg fusion and is not required for the acrosome reaction. To test whether IVF failure with cumulus-intact and cumulus-free oocytes is due to the inability of the KO sperm to penetrate the zona pellucida, we pre-treated oocytes with CARD medium. CARD medium contains reduced glutathione that has been shown to loosen and expand the zona pellucida [27]. After treating oocytes with CARD medium, we observed a partial rescue of fertilization, with an average fertilization rate of 37% (*n* = 3). The partial rescue suggests IVF failure with cumulus-intact and cumulus-free oocytes is due to a failure of sperm to penetrate the zona pellucida, which further implicates reduced motility as explaining the subfertility seen in the *Tcp11* KO males. To examine whether TCP11 functions in sperm capacitation, tyrosine phosphorylation [28–31] was examined in sperm incubated in TYH medium after 10 and 120 min. Western blot analysis using an anti-tyrosine phosphorylation antibody detects several bands in sperm proteins from wild type and *Tcp11*-null sperm (Supplementary Figure S5), suggesting that capacitation may have occurred in null sperm.

### TCP11 is expressed in the late stages of spermiogenesis and not present in mature sperm

**Figure 6:**
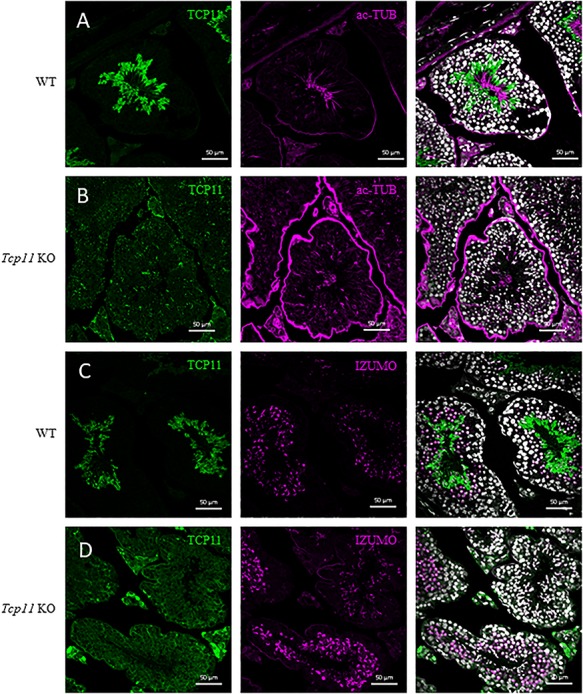
TCP11 localizes to the cytosol in late spermiogenesis. (A and B) Anti-TCP11 antibody (green) displays cytoplasmic localization in testis cross sections from wild-type mice. In Tcp11 KO testis cross sections, anti-TCP11 antibody staining (green) is punctate and present in all cell types. Anti-acetylated-TUBULIN (magenta) localizes to the flagellum of spermatids in both wild-type and KO cross sections. White = Hoechst 33342. Scale bars = 50 μm. (C and D) Anti-TCP11 antibody (green) displays cytoplasmic localization in testis cross sections from wild-type mice. In Tcp11 KO testis cross sections, anti-TCP11 antibody staining (green) is diffuse in the testis and brightly stains the interstitial cells. Anti-IZUMO1 (magenta) localizes to the developing acrosome in round and elongated spermatids in both wild-type and KO cross sections. White = Hoechst 33342. Scale bars = 50 μm.

**Figure 7:**
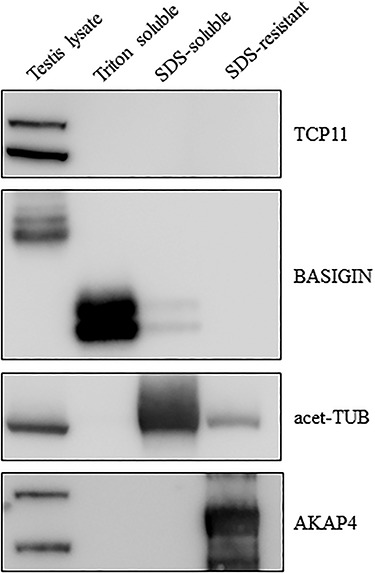
TCP11 appears to not be present in mature sperm. Sperm isolated from the cauda epididymis were used for protein extraction into a Triton X-100- (membrane bound and cytoplasmic soluble), SDS- (axonemal components), and SDS-insoluble (fibrous sheath and outer dense fibers) pool. Western blot analysis against BASIGIN, acetylate-Tubulin, and AKAP4 demonstrates successful extraction of these three pools, respectively. Anti-TCP11 antibody only detects bands in the wild-type testis lysate.

To determine the localization of TCP11, we used our anti-TCP11 antibody to perform immunofluorescence on testis sections from control or *Tcp11* KO males. To visualize the acrosome or flagellum, we stained with anti-IZUMO1 or anti-acetylated-TUBULIN antibody, respectively. In wild-type testis-sections, the TCP11 antibody signal appears to localize to the cytoplasm in the late steps of spermatid development (step 15, Supplementary Figure S6) and does not colocalize with anti-IZUMO1 or anti-acetylated-TUBULIN signal (Figure 6A and C and Supplementary Movies 7–9). The staining pattern indicates that TCP11 is a cytoplasmic protein, which is consistent with SignalP and transmembrane predictions of TCP11 as a non-secreted, non-transmembrane containing protein. In KO sections, there was little signal or what appears to be background staining from the anti-TCP11 antibody (Figure 6B and D). Our staining results are consistent with the previously reported localization of TCP11 [20]. To determine whether TCP11 may localize in mature sperm, we fractionated sperm extracted from the cauda epididymis. Sperm proteins were fractionated into a Triton X-100-, SDS-, and insoluble fraction corresponding to the membrane bound/cytoplasmic soluble, axonemal proteins, and fibrous sheath, respectively [32, 33]. Western blot analysis of fractionated sperm proteins does not detect the presence of TCP11 with our anti-TCP11 antibody (Figure 7) or with the previously published antibody (Supplementary Figure S7) [20]. Western blot analysis and immunofluorescence indicates that TCP11 function is restricted to the cytoplasm in late stage spermatids and appears to be absent in mature sperm.

### *Tcp11*-null sperm have decreased PKA activity

**Figure 8:**
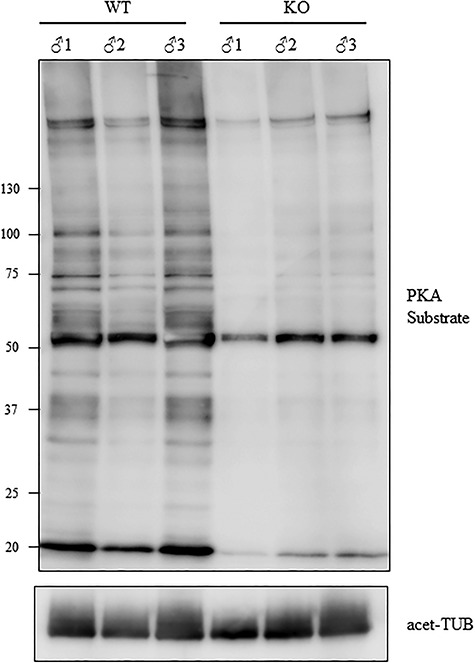
Tcp11 KO sperm have decreased phosphorylation of PKA substrates. Western blot analysis using anti-PKA substrate phosphorylation antibody shows decreased signal in sperm proteins isolated from three Tcp11 KOs. Sperm were incubated for 10 min in TYH medium prior to protein extraction.

Previous reports implicated TCP11 in cAMP signaling by forming a complex with PKA in sperm [23, 34]. While we could not detect TCP11 in sperm, we wanted to determine whether PKA signaling was affected in the absence of TCP11. Sperm from the cauda epididymis from three wild-type and *Tcp11* KO males were isolated into TYH media and incubated for 10 min at 37 °C with 5% CO_2_. Sperm proteins were then isolated after the incubation and subjected to Western blot analysis using an antibody that recognizes the phosphorylated target motif of PKA (R-R-X-S/T-X). Western blot analysis revealed decreased signal from the *Tcp11* KO samples when compared to the wild-type controls (Figure 8). It should be noted, while there is variability between the individual males with one male displaying higher signal in the Western blots, the overall trend is reduced signal compared to wild-type males. This suggests that phosphorylation of PKA targets is reduced, which further suggests that PKA signaling is reduced in the *Tcp11* KO. These results are consistent with previous reports implicating TCP11 in cAMP/PKA signaling [23, 34].

## Discussion

We show that mouse *Tcp11* is an evolutionary-conserved, testis-enriched gene that is expressed from day 15 when the leading edge of spermatogenesis enters the pachytene stage. Previous reports on mouse *Tcp11* also showed mRNA expression in spermatocytes [20]. *Tcp11* localizes to the distal end of the *t*-complex on chromosome 17. Interestingly, *Tcp11* has undergone duplication with paralogs *Tcp11l1*, *Tcp11l2*, and *Tcp11l3* present on chromosome 2, 10, and X, respectively. Of these paralogs, only *Tcp11l3* shows testis-specific expression, while *Tcp11l1* and *Tcp11l2* show broad expression in multiple tissues. The wider expression pattern of *Tcp11* genes suggests that these genes play roles in other tissues besides the testis. Unlike mouse, human *TCP11* shows testis-enriched expression and low-level expression in brain and epididymis, which suggests that human TCP11 has functions in these tissues also. Two variant transcripts of mouse *Tcp11* are annotated with the longer version encoding 567 amino acids and the shorter encoding 488 amino acids. Both the longer and shorter version of TCP11 contains a conserved domain that is specific to *Tcp11* homologs and is called the TCP11 domain. This domain has no known catalytic function; however, it has some homology (25% identity and 49% similarity) to the yeast protein SOK1 [35]. In addition to the two annotated *Tcp11* variants, another two variants of TCP1 are predicted to exist (454 and 447 amino acids). Western blot analysis detected what appears to be a doublet running slightly larger than 50 kD, which could correspond to these additional TCP11 variants. There is a fifth band specific to the wild type, migrating at less than 50 kD, and whose identity is unknown. It is possible that this band may be a degradation product of a longer version of TCP11; however, this fifth band is very weak compared to the other four variants.

In our studies, we show that a null mutation in *Tcp11* using a Knockout Mouse Project (KOMP) line results in severe subfertility in males which sired few pups when mated with wild-type females over a three-month period. No obvious defect was detectable through histology of testes from the KO males, and in addition, immunofluorescence of testis cryosections did not reveal defects in spermatogenesis. However, sperm from the KO males revealed abnormal morphology and decreased motility when analyzed via CASA. A previous report has correlated human TCP11 with sperm morphology, where sperm from infertile men that display aberrant morphology has less TCP11 [36], and proteomics analysis identified TCP11 in the flagellum of human sperm [37]. Proteomics analysis of rat sperm also identified TCP11 [38]. However, as we have shown in this report, mouse sperm from the epididymis does not contain detectable levels of TCP11 by Western blot, and a proteomic analysis of mouse sperm proteins did not identify TCP11 [39]. Perhaps mouse sperm differs from human and rat sperm by removing TCP11 from maturing spermatozoa. Databases of human variants list up to 8000 variants in TCP11; however, no clinical significance in sperm function has been attributed to any variant as of this publication. As would be expected from decreased sperm motility, sperm from KO animals had difficulty fertilizing cumulus-intact and cumulus-free oocytes but were successful with zona pellucida-free oocytes. By loosening the zona pellucida by pre-treating eggs with CARD medium, we partially rescued the fertilization defect with KO sperm. Our IVF data indicate that TCP11 functions in sperm motility either directly or indirectly by activation of the cAMP/PKA pathway, since Tcp11-null sperm has decreased PKA activity.

Localization of TCP11 was initially reported to be restricted to the late stage spermatids and absent from mature sperm [20]. We generated an antibody to the N-terminus of the two verified TCP11 variants (62 and 54 kD isoforms) from the Ensembl database and were able to detect these variants in wild-type testis lysates migrating close to their predicted size using Western blot analysis. These bands disappeared when we probed testis lysates from KO animals, both confirming the specificity of our antibody and confirming that our KO mice are true protein nulls. Immunoblots from proteins isolated from the epididymis under harsher buffer conditions (RIPA buffer with 5 mM DTT) did not detect TCP11 but readily detect the acrosomal membrane ligand IZUMO1. In addition, immunofluorescence staining with our antibody revealed that TCP11 localized to the cytoplasm of late stage spermatids and showed no colocalization with an acrosome marker (IZUMO1) or a flagellum marker (acetylated-Tubulin). Fractionation of epididymal sperm proteins into membrane/cytoplasmic proteins, axonemal proteins, and the fibrous sheath also did not detect TCP11 when analyzed using Western blots. The previously published antibody of TCP11 [20, 21] raised against full length TCP11 showed additional variants of TCP11, two extra that were predicted by the Ensembl database running as a doublet slightly larger than 50 kD and possibly a fifth running below 50 kD. Like our anti-TCP11 antibody, this previously published antibody did not detect any TCP11 variants in epididymal lysates or fractionated sperm proteins. Our studies on TCP11 localization and fractionation are in direct conflict with previously published reports showing TCP11 present on the sperm surface [21, 23, 34]. It is unclear why there is a discrepancy; one possibility is that there might be spurious binding of primary antibody to the sperm surface [40]. SignalP and transmembrane prediction algorithms do not detect a signal sequence or a transmembrane region in TCP11. Without a KO control, it would be difficult to interpret the binding of antibodies to the sperm surface in the previous studies [21, 23, 34].

Our data indicate a major role of TCP11 in sperm motility; however, since TCP11 is only present in elongating spermatids and absent from mature sperm in mouse, it is unclear how TCP11 role in spermatids is affecting motility in mature sperm where it is not present. TCP11 has some homology with the *Saccharomyces cerevisiae* protein Sok1, which has been implicated in PKA signaling [35, 41, 42]; however, Sok1 is a nuclear localized protein, while mouse TCP11 is cytoplasmic. Further, the molecular mechanism by which Sok1 functions in PKA signaling is still unclear. In *Candida albicans*, Sok1 is involved in hyphal growth and functions independently of the PKA signaling pathway [43, 44]; however, like Sok1 in *S. cerevisiae*, the mechanisms of Sok1 function is yet to be determined. It should be noted that while there is a decrease in phosphorylation of PKA targets in the Tcp11 KO, the activity of PKA phosphorylation is not completely abolished. One possibility is that the other TCP11 paralogs can partially compensate, as several of them are expressed in the testis. Examining the mechanism of TCP11 function is complicated that several variants of TCP11 exist; however, with antibodies recognizing the TCP11 variants, perhaps it will be possible to tease out the mechanism of how TCP11 functions in the PKA pathway.

## Materials and methods

### Animals

*Tcp11* ^tm1a(KOMP)Wtsi/+^ (referred to as *Tcp11^fl/+^*) mice were obtained from the KOMP consortium. The null allele was generated using JM8A3.N1 ES cells, a pD223-DTA plasmid backbone, and homology arms 5.8kB upstream and 3.7kB downstream of *Tcp11* (MGI: 98544). *Tcp11^fl/+^* were crossed with *Gdf9*-iCre to generate *Tcp11^−/+^* mice. *Tcp11* mutant mice were maintained on a mixed genetic background (C57B6/129SvEv or B6D2F1), while wild-type mice were purchased from CLEA Japan (Tokyo, Japan) or Japan SLC (Shizuoka, Japan) or were produced from intercrosses of C57BL6 and 129S6/SvEv mice generated in the Matzuk laboratory at Baylor College of Medicine. All mice were housed in specific pathogen free animal facilities with a light:dark cycle of 12:12. All animals in this study were approved by the Institutional Animal Care and Use Committees of Baylor College of Medicine (Houston, TX) and the Research Institute for Microbial Diseases of Osaka University (Osaka, Japan).

### Reverse transcription polymerase chain reaction

Mouse cDNA was prepared from multiple adult tissues of B6D2F1 and C57CL6/129SvEv hybrid mice and testes of 5 to 60-day-old mice. Human cDNAs were obtained from the Human Tissue Acquisition and Pathology core service (Baylor college of Medicine, USA). The primers and amplification conditions for each gene are summarized in Supplementary Table S1.

### Sequence comparisons

Sequence comparisons between Tcp11 homologs were done with BLAST or with Clustal Omega.

### Mating tests

Five heterozygous (*Tcp11^+/−^*) and *Tcp11^−/−^* sexually mature males were paired with 6-week-old wild-type females for three months. The number of pups delivered was counted the day after birth.

### Western blot analysis

Testis and epididymides were dissected from wild-type and *Tcp11^−/−^* adults and placed into PBS. Testis samples were lysed in 1 mL RIPA (50 mM Tris pH 7.5, 150 mM NaCl, 1 mM DTT, 1% Nonident P-40, 0.5% deoxycholate, 0.1% SDS, protease inhibitors) using Qiagen’s TissueLyser II (30 Hz, 2 min, RT). Epididymides were first minced with dissecting scissors in 1 mL RIPA (50 mM Tris pH 7.5, 150 mM NaCl, 5 mM DTT, 1% Nonident P-40, 0.5% deoxycholate, 0.1% SDS, protease inhibitors) and then lysed with Qiagen’s TissueLyser II (30 Hz, 2 min, RT). Lysates were clarified with a 10 min spin at 18 000×*g* at 4 °C. The protein concentration was determined with the Bradford method. Thirty microgram per sample were loaded per well. Western blot analysis was performed using BioRad’s TransBlot Turbo. PVDF membranes were blocked with TBS with 0.05% Tween and 5% milk and washed with TBS with 0.05% Tween and 0.5% milk. Both primary and secondary antibodies were diluted in washing buffer. Nacalai’s Super Signal was used for chemiluminescence and detected with Image Quant. Antibodies used include the following: goat anti-BASAGIN 1:1000 (Santa Cruz), mouse anti-AKAP4 1:1000 (BD Biosciences), and mouse anti-Acetylated Tubulin 11 000 (Sigma).

### Isolating sperm proteins for tyrosine phosphorylation and PKA target phosphorylation analysis and sperm fractionation

For analyzing tyrosine phosphorylation, sperm were incubated in TYH medium for 10 min or 2 h. For PKA target phosphorylation, sperm were incubated in TYH medium for 10 min. Sperm were then collected in 1 mL PBS and centrifugated at 2000*×g* for 2 min at room temperature. The collected sperm were lysed with sample buffer (66 mM Tris-HCl, 2%, SDS, 10% glycerol and 0.005% Bromophenol Blue), boiled for 5 min, and centrifugated at 15 000*×g* for 15 min at 4 °C. For Western blot analysis, 5 μL per sample were loaded per well. Blotting was done using BioRad’s TransBlot Turbo. PVDF membranes were blocked with TBS with 0.05% Tween and 5% BSA and washed with TBS with 0.05% Tween and 0.5% BSA. Millipore’s 4G10 monoclonal antibody against phosphorylated tyrosine and Cell Signaling’s 100G7E antibody against PKA substrate were used at 1:1000 for Western blot analysis.

For sperm fractionation, sperm were extracted from the cauda epididymis from wild-type males (B6D2F1) into TYH media. Sperm were centrifuged at 2000*×g* for 2 min at room temperature. The sperm pellet was then resuspended in 100 μL of Triton X-100 extraction buffer (20 mM Tris pH 7.5, 50 mM NaCl, 1% Triton X-100, and Protease Inhibitors) and incubated for 2 h at 4 °C with gentle mixing. The sample was centrifuged for 10 min, 4 °C, 15 000*×g*. The pellet was resuspended in 100 μL SDS extraction buffer (75 mM NaCl, 24 mM EDTA, 1% SDS) and incubated at room temperature for 1 h with gentle mixing. The sample was then centrifuged for 10 min, 4 °C, 15 000*×g*. The pellet was resuspended in 100 μL 1X SDS PAGE sample buffer. For SDS PAGE, 1 μL per sample was loaded per well.

### TCP11 antibody production

Sigma’s antibody production service was utilized to produce our TCP11 antibody. The peptide sequence AAESASRESRGGNTRESA corresponding to amino acids 15–32 of mouse TCP11 was used to immunize one rabbit. After 54 days, the blood serum was extracted. Anti-TCP11 antibodies were purified using an affinity resin (Sulfo-Link) generated with the immunizing peptide. The previously published anti-TCP11 antibody was a kind gift from [20, 21]. Both antibodies were used at 1:1000 dilution for Western blot analysis. The Novus anti-TCP11 antibody (NBP1-57698) was used at 1:500 dilution for Western blot analysis [23].

### Histology

For PAS staining, testis and epididymides were fixed in Bouin’s fixative, processed, and embedded in paraffin. Sections were cut at 5 μm thickness. Sections were then deparaffinized, rehydrated, stained with Periodic Acid-Schiff’s Reagent, counterstained with hematoxylin, dehydrated, and mounted with VectaMount or Permount.

### In vitro fertilization

Seven-week-old B6D2F1 females were injected with 0.1 cc HyperOva. Forty-eight hours later, the females were injected with 0.2 cc hCG. The next day oviducts were collected, and cumulus-oocyte-complexes were isolated into TYH medium or CARD medium. CARD medium was prepared according to manufacturer’s instructions. The glutathione concentration recommended for IVF using frozen-thawed sperm was used for the CARD medium. Some eggs in TYH medium were treated with hyaluronidase to generate cumulus-free oocytes, while another batch was treated with collagenase to generate zona pellucida-free oocytes. Sperm from wild-type and *Tcp11*^*−/*−^ males was isolated and incubated in TYH medium at 37 °C with 5% CO_2_ for 2 h. After the sperm incubation, 2x10^5^ sperm/mL was used for fertilization. Four hours after incubation, zygotes in CARD medium were washed with KSOM medium [45] and incubated until the next day. Six hours after incubation, zygotes with two or more pronuclei were counted from the zona-free condition. The next day, 2-cell embryos which developed in the cumulus intact, cumulus free, and CARD medium conditions were counted.

### Sperm analysis

Sperm motility was analyzed as previously described [46]. Sperm from wild-type and *Tcp11^−/−^* mice was extracted into TYH media and incubated at 37 °C with 5% CO_2_. CASA (Hamilton Thorne) with CEROS I software was used to measure sperm motility after 10 min and 120 min incubation.

### Immunofluorescence

For testis cryosections, immunostaining was performed as previously described [47]. Primary antibodies used include: mouse anti-AcetTubulin 1:1000 (Sigma), rat anti-IZUMO1 1:200 [48], and rabbit anti-TCP11 1:500. Secondary antibodies used include: Alexa Fluor 488 anti-rabbit, Alexa Fluor 5465 anti-mouse, and Alexa Fluor 546 anti-rat used at 1:1000.

### Statistical analysis

Statistical analysis was done when three or more males were used for experiments. Fisher’s exact test or Student’s *t*-test were used to examine statistical significance. *P*-values between 0.05 and 0.001 were considered significant (^*^), while *P*-values below 0.001 were considered highly significant (^**^).

## Supplementary Material

Supplementary_ioz226
