# Supplementary figures and images for: Mouse t-complex protein 11 is important for progressive motility in sperm
Source: Biol Reprod. 2019 Dec 14;102(4):852–62. doi: 10.1093/biolre/ioz226 (PMC7124965; doi:10.1093/biolre/ioz226)

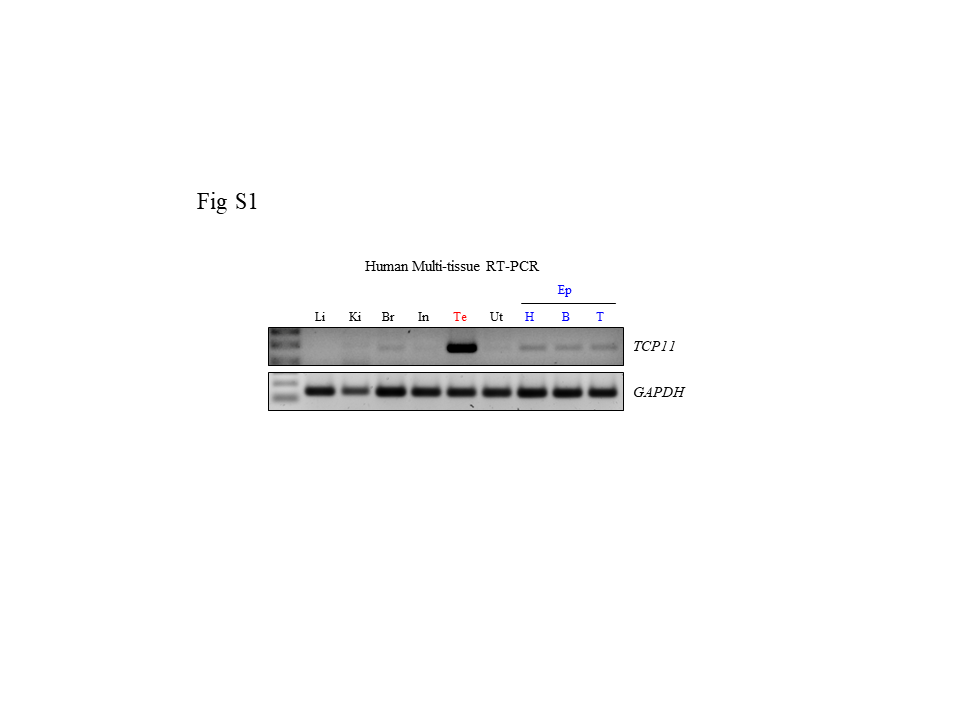

Supplement: Supplementary_ioz226 [file supplementary_ioz226.zip › Supplementary_ioz226/Supplementary Figure 1.tif]

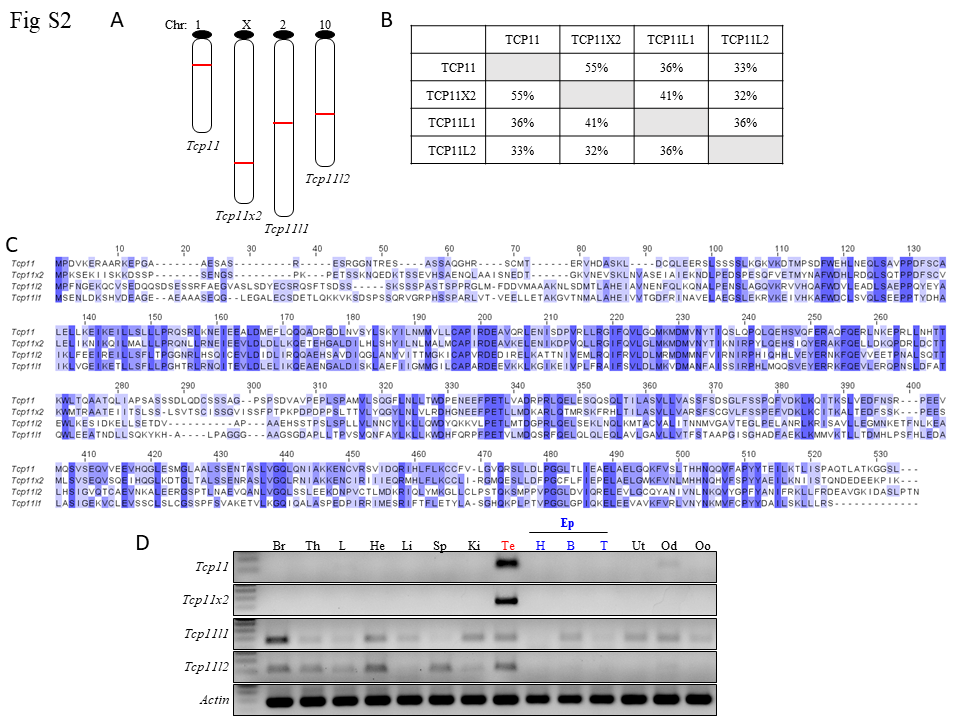

Supplement: Supplementary_ioz226 [file supplementary_ioz226.zip › Supplementary_ioz226/Supplementary Figure 2.tif]

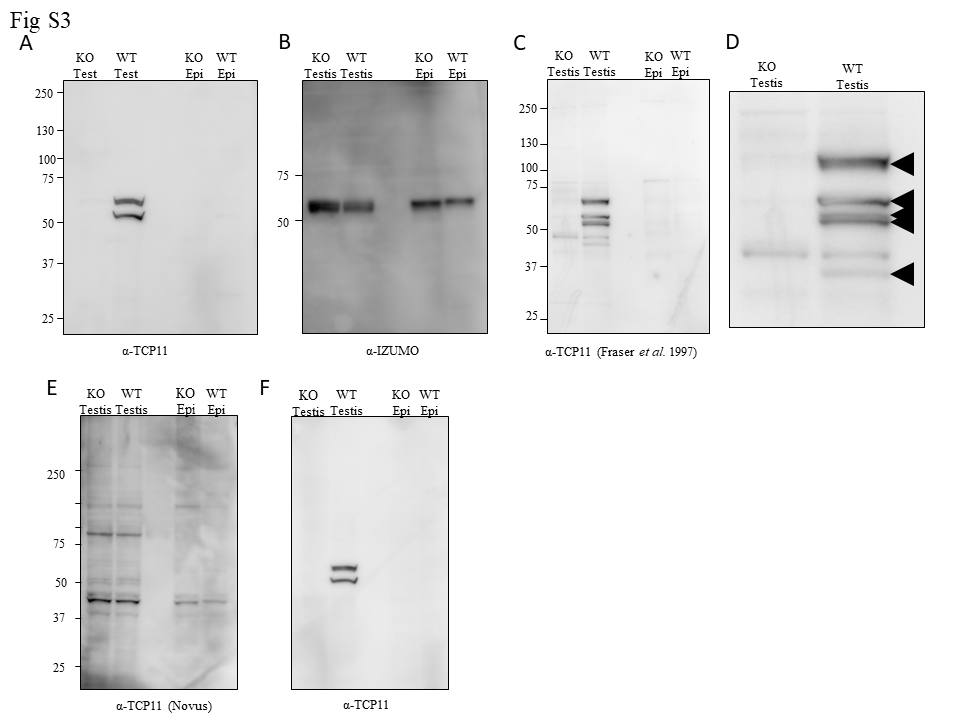

Supplement: Supplementary_ioz226 [file supplementary_ioz226.zip › Supplementary_ioz226/Supplementary Figure 3.tif]

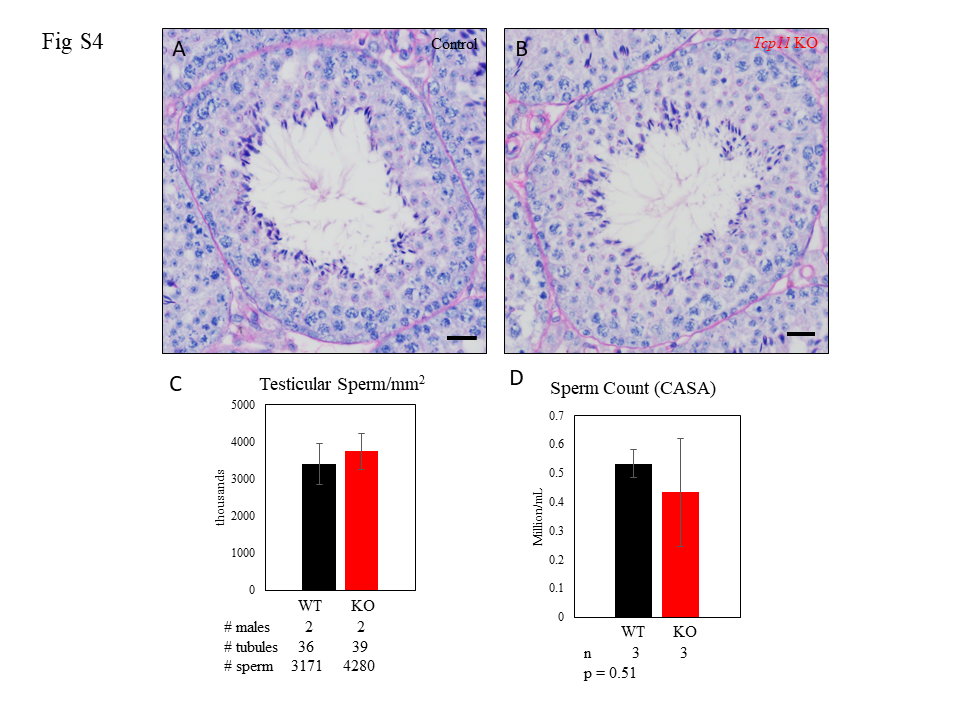

Supplement: Supplementary_ioz226 [file supplementary_ioz226.zip › Supplementary_ioz226/Supplementary Figure 4.tif]

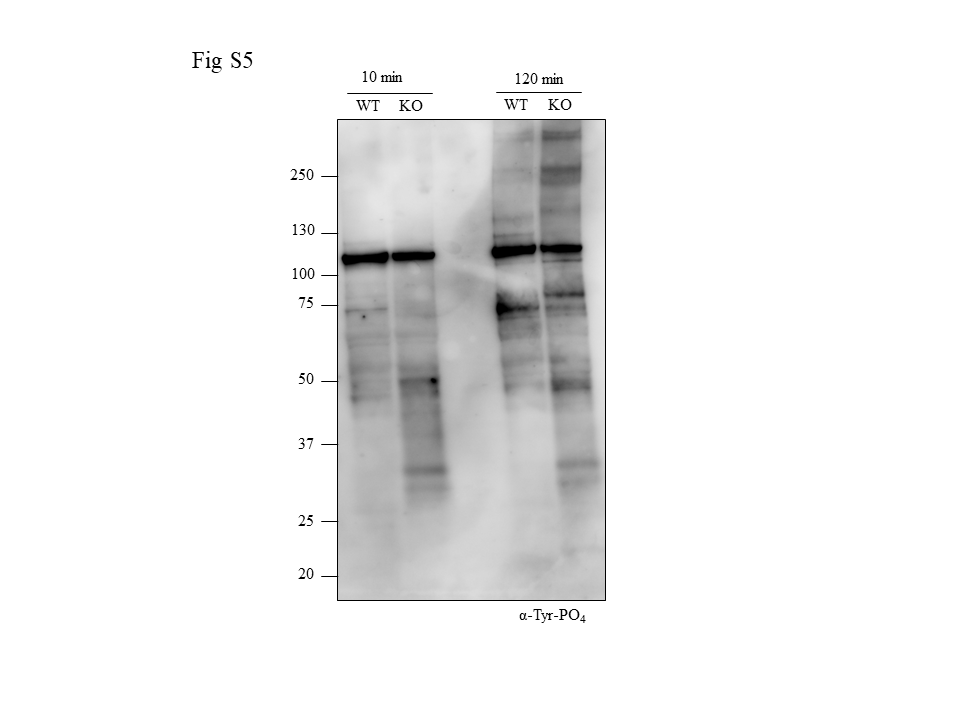

Supplement: Supplementary_ioz226 [file supplementary_ioz226.zip › Supplementary_ioz226/Supplementary Figure 5.tif]

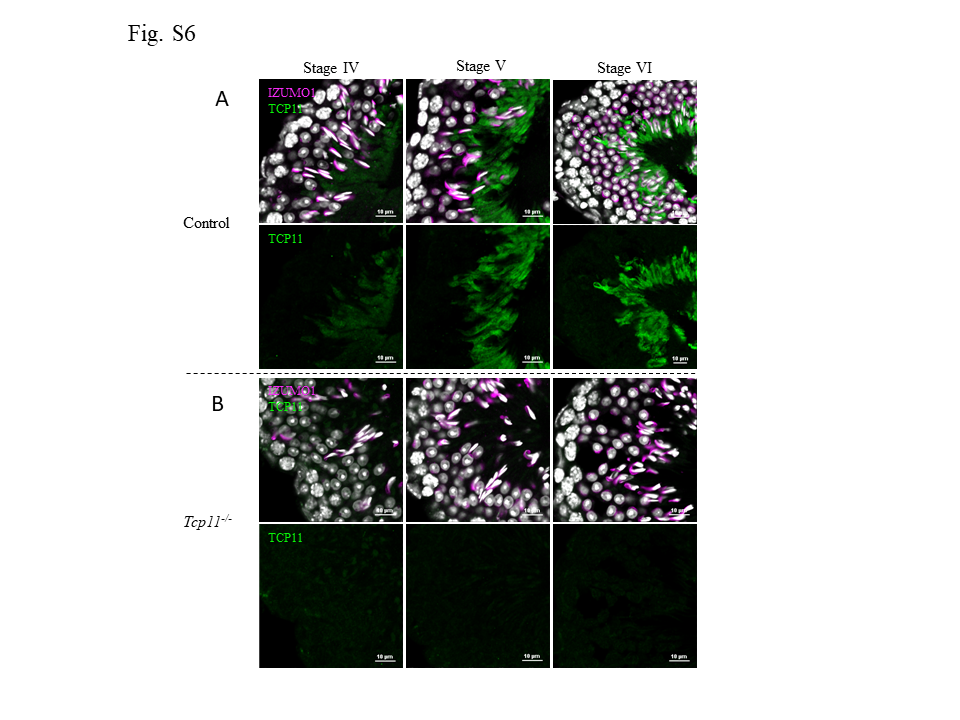

Supplement: Supplementary_ioz226 [file supplementary_ioz226.zip › Supplementary_ioz226/Supplementary Figure 6.tif]

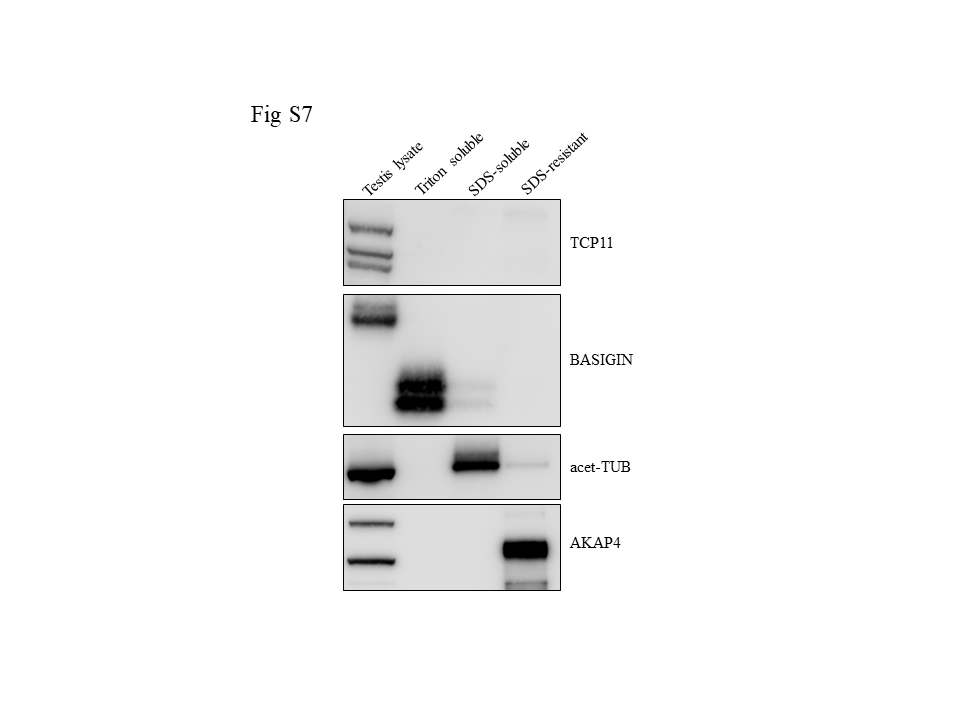

Supplement: Supplementary_ioz226 [file supplementary_ioz226.zip › Supplementary_ioz226/Supplementary Figure 7.tif]
